# Comparative effectiveness of dienogest versus ethinyl estradiol/levonorgestrel combined oral contraceptive therapy in women with endometriosis-associated chronic pelvic pain

**DOI:** 10.3389/fendo.2026.1884571

**Published:** 2026-08-25

**Authors:** Bushra Bahar, Neena Gupta, Alok Raghav, Uruj Jahan, Rashmi Yadav

**Affiliations:** 1 Department of Obstetrics and Gynecology, GSVM Medical College, Kanpur, India; 2 Associate Professor, Department of Biotechnology, Rama University Rama City, Mandhana, Kanpur, Uttar Pradesh, India; 3 Adjunct Faculty, University Centre for Research & Development (UCRD), Chandigarh University, Chandigarh, India; 4 Adjunct Faculty, Center for Global Health Research, Saveetha Medical College and Hospital, Chennai, India

**Keywords:** dienogest, endometriosis, oral contraceptives, pain, visual analog scale

## Abstract

**Objective:**

To compare the clinical effectiveness of combined oral contraceptive pills (levonorgestrel-containing COC) and dienogest in the management of endometriosis-associated chronic pelvic pain and related symptoms.

**Methods:**

This prospective observational cohort study was conducted in the Department of Obstetrics and Gynecology at GSVM Medical College, Kanpur, India, between 2023 and 2024. A total of 140 women with clinically and ultrasonographically diagnosed endometriosis-associated pelvic pain completed the study and were included in the final analysis. Participants received either dienogest (2 mg once daily) or a continuous combined oral contraceptive containing ethinyl estradiol 0.03 mg and levonorgestrel 0.15 mg for 3 months. Pain severity was assessed using the Visual Analog Scale (VAS) at baseline and during monthly follow-up visits. Longitudinal changes in pain scores and associated symptoms were compared between the treatment groups.

**Results:**

Baseline demographic and clinical characteristics were comparable between the groups. The mean age of participants in the OCP and dienogest groups was 31.47 ± 8.90 and 31.08 ± 8.78 years, respectively (p=0.794). Baseline mean VAS scores were 8.55 ± 0.98 and 7.77 ± 0.90 in the OCP and dienogest groups, respectively. A significant reduction in VAS scores was observed in both groups throughout the follow-up period (p<0.001). At 3 months, the mean VAS score decreased to 4.28 ± 1.15 and 5.22 ± 1.66 in the OCP and dienogest groups, respectively (p<0.001). Significant improvement in dysmenorrhea, dyspareunia, menstrual irregularities, and chronic pelvic pain was also observed in both groups during follow-up.

**Conclusion:**

Both treatments were associated with significant improvement in endometriosis-associated pain, and the OCP group showed greater short-term pain reduction in this cohort, suggesting that the evaluated levonorgestrel/ethinyl estradiol regimen may represent a conservative treatment option, particularly in resource-limited healthcare settings. Larger multicenter longitudinal studies are warranted to further validate these findings.

## Introduction

Endometriosis is a chronic, estrogen-dependent inflammatory disorder characterized by the presence of endometrial-like tissue outside the uterine cavity. It commonly presents with chronic pelvic pain, dysmenorrhea, dyspareunia, and infertility (1–3). A definitive diagnosis of endometriosis requires laparoscopy with ablation or excision of affected tissue. However, post-surgical endometriosis recurrence is common and poses a limitation during its management. A prospective cohort study demonstrated that women undergoing hysterectomy with ovarian preservation had nearly twice the risk of ovarian failure compared to women with an intact uterus (HR 1.92, 95% CI 1.29–2.86), with an estimated 14.8% developing ovarian failure within 4 years versus 8.0% of controls (4).

The UK National Institute for Health and Care Excellence and the European Society of Human Reproduction and Embryology have recommended levonorgestrel-containing COC, along with progestogens, for the management of endometriosis-related pain (5). Endometriosis is a chronic, estrogen-dependent, inflammatory gynecological disorder characterized by the presence of endometrium-like tissue at extra-uterine sites (6). It affects approximately 10% of women of reproductive age worldwide and constitutes a leading cause of chronic pelvic pain, dysmenorrhea, dyspareunia, infertility, and overall impairment of reproductive health (7). Many detrimental effects are associated with the disorder, affecting not only physical and psychological well-being but also social and occupational performance, thereby reducing the quality of life. Despite advances in diagnosis and medical management, delayed diagnosis, symptom recurrence, and long-term disease management remain major clinical challenges in women with endometriosis (8).

The pathophysiology of endometriosis involves several interacting factors, including estrogen-induced hyperproliferation, chronic inflammation, immune dysfunction, angiogenesis, and progesterone resistance (9). Abnormal expression of estrogen receptor beta (ER-beta) increases local aromatase activity and proinflammatory cytokine levels, which are responsible for the development of endometrial tissue lesions (10). Inflammation results in sensitization of pelvic pain pathways, causing chronic pain and decreased quality of life. Since endometriosis is a chronic condition, medical management is crucial to alleviate symptoms and slow disease progression (10).

Hormonal treatment is the mainstay of management for pain associated with endometriosis in adolescents. Levonorgestrel-containing COC are commonly used because they inhibit ovulation, reduce menstrual bleeding, limit endometrial growth, and reduce prostaglandin-induced inflammation (11). Studies have demonstrated that continuous OCP administration leads to improvements in dysmenorrhea and chronic pelvic pain among affected individuals. However, the use of levonorgestrel-containing COC faces various problems, including continual exposure to estrogen, breakthrough bleeding, recurrence of symptoms upon withdrawal, and individual variations in response (11).

Oral selective progesterone receptor modulator, dienogest, which has antiproliferative and anti-inflammatory effects, has proven to be a promising drug for the long-term treatment of endometriosis (12). Dienogest prevents ovulation, inhibits local estrogen production, induces decidualization and atrophy of endometriotic lesions, and reduces inflammatory cytokine levels. Various trials have shown that the use of dienogest is associated with significant relief of pelvic pain and dysmenorrhea, as well as better quality-of-life scores in patients with moderate-to-severe endometriosis (12).

Despite the widespread clinical use of both dienogest and combined levonorgestrel-containing COC, comparative effectiveness data remain heterogeneous across populations. The varied designs, short follow-up periods, and inconsistent pain assessment methods limit many studies. Comparative real-world evidence from Indian cohorts is particularly limited. To address this gap, we conducted the present prospective observational cohort study to compare the clinical effectiveness of dienogest versus continuous combined levonorgestrel-containing COC for the reduction of endometriosis-associated chronic pelvic pain and related symptoms among women managed conservatively.

## Materials and methods

### Ethics statement

This study was conducted in accordance with the ethical principles of the Declaration of Helsinki. This study was approved by the Institutional Ethics Committee of GSVM Medical College, Kanpur (Ethics Approval Number: EC/181/July/2023, dated 05/07/2023). Written informed consent was obtained from all participants before enrolment after the objectives, procedures, benefits, and possible adverse effects of the study interventions were explained.

### Study design and setting

This prospective observational cohort study was conducted from 2023 to 2024 at the Department of Obstetrics and Gynecology, GSVM Medical College, Kanpur, India. This experimental study aimed to determine the effect of dienogest and levonorgestrel-containing COC on endometriosis-related pain in female volunteers. The final analyzed sample size provided adequate statistical power to detect clinically meaningful differences in VAS score reduction between the groups; the computed sample size at a 95% confidence interval was 70 per study group.

All study participants with endometriosis-related pelvic pain confirmed via USG who met the following inclusion criteria were enrolled: (i) willingness to provide written informed consent; (ii) female patients aged between 18 and 45 years at the time of consent; (iii) a documented diagnosis of endometriosis with pelvic pain confirmed by USG; and (iv) women of childbearing potential with a negative urine pregnancy test before study entry who agreed to comply with contraception requirements. All [Note: Women of childbearing potential are defined as those who have not undergone surgical sterilization, such as bilateral oophorectomy, hysterectomy, or bilateral tubal ligation.] Endometriosis was diagnosed through clinical examination and pelvic ultrasound. Ultrasound findings that could be characteristic of endometriosis, such as ovarian endometriomas, were documented when present. However, routine histopathological confirmation and standardized laparoscopic staging were not available for all participants.

The following individuals were excluded from the study: (i) those with chronic pelvic pain not attributed to endometriosis, which was defined as intermittent or constant noncyclic pelvic pain persisting for at least six months, with or without associated symptoms such as dysmenorrhea, dyspareunia, dysuria, or dyschezia; (ii) a clinically established need for primary surgical treatment of endometriosis; (iii) known or suspected pregnancy; (iv) lactating women; (v) individuals planning to conceive during the study period; (vi) those with functional ovarian cysts; (vii) individuals with uncontrolled hypertension (SBP >160 mmHg or DBP >100 mmHg); (viii) those with active venous thromboembolic disorders; (ix) a current or prior history of arterial or cardiovascular disease, including congenital heart failure, arrhythmias, stroke, or congenital heart disease; (x) HbA1c levels >9.0%; (xi) severe renal impairment (eGFR <30 ml/min/1.73 m²); (xii) vascular complications resulting from diabetes mellitus; (xiii) severe hepatic disease (chronic: Child-Pugh C; acute: fatty liver grade >1 and INR >1.5); (xiv) a history of severe hepatic disease with abnormal liver function; (xv) the presence or history of benign or malignant liver tumors; (xvi) known or suspected malignancies; (xvii) undiagnosed abnormal vaginal bleeding; (xviii) ophthalmic vascular disease; (xix) a current or prior history of migraine with focal aura; (xx) osteoporosis or, in the investigator’s opinion, an increased risk of osteoporosis; and (xxi) individuals who smoked and were unable or unwilling to cease smoking during the study. Women were eligible regardless of marital status or parity, provided they met the clinical and ultrasonographic criteria for endometriosis-related pelvic pain. Ovarian reserve markers, such as AMH and AFC, were not routinely measured or used for exclusion. Participants underwent clinical assessment and ultrasound evaluation to identify endometriosis-associated pelvic pain, and those with clinically apparent alternative causes of pelvic pain were excluded from the study. Previous exposure to hormonal therapy for endometriosis (including GnRH agonists, progestogens, or combined oral contraceptives) was not systematically documented as part of the study protocol; therefore, it was not incorporated into baseline matching.

### Participants enrollment

**Figure 1:**
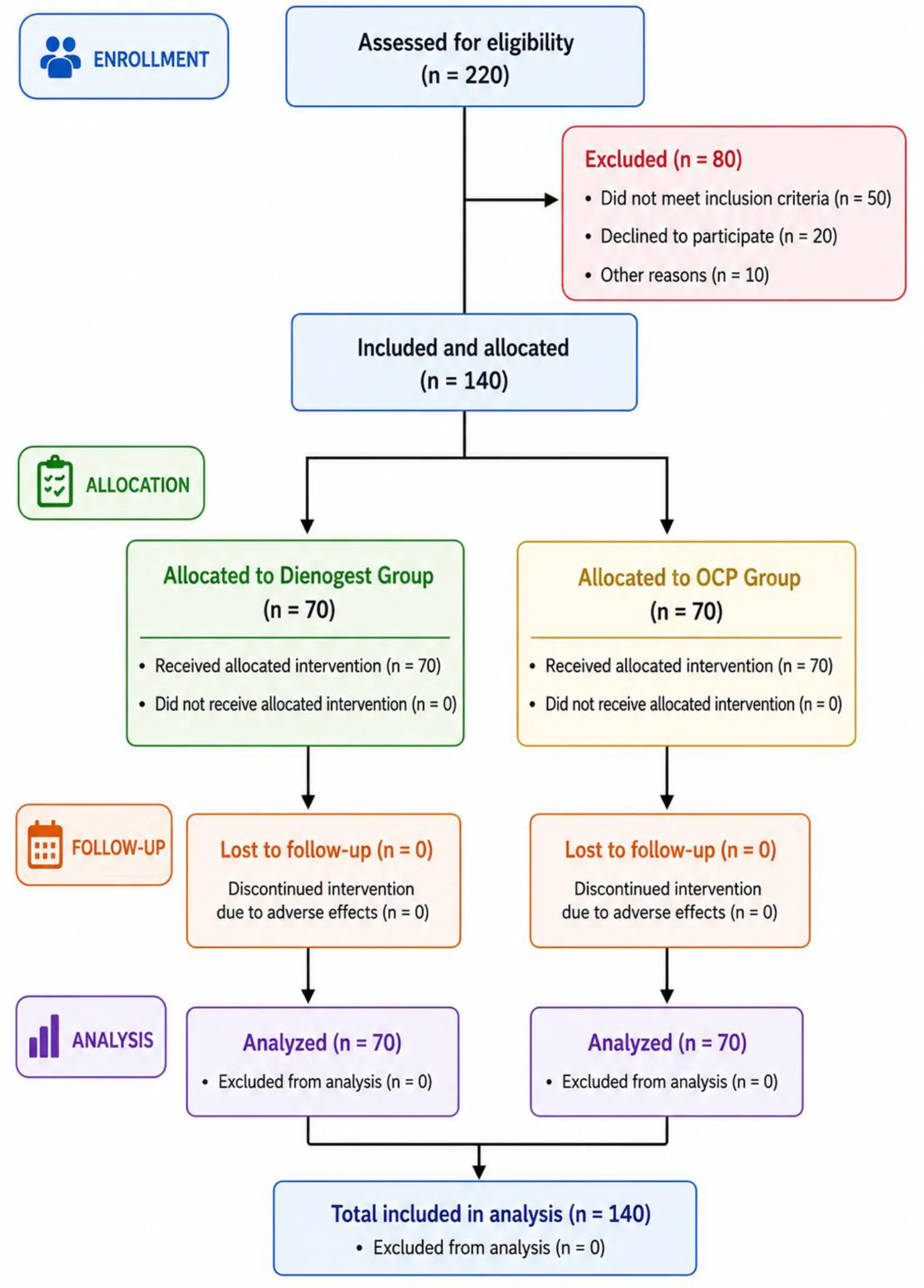
Study participant flow diagram.

A total of 220 eligible participants who met the inclusion criteria were assigned to two study groups: 108 participants in Group I and 112 participants in Group II. Furthermore, almost 80 participants were lost to follow-up or discontinued the intervention in both groups; hence, 70 participants per group were included in the outcome analysis. The baseline demographic characteristics of the participants lost to follow-up were comparable to those included in the final analysis. The participants were managed according to routine clinical practice. After counseling on treatment options, the choice between dienogest and continuous combined oral contraceptive therapy was made based on clinician judgment and patient preference, considering contraindications and individual clinical circumstances. All participants were examined by a designated gynecologist. Group 1 received dienogest 2 mg orally once daily for 90 days, while Group 2 received continuous low-dose oral contraceptive therapy (ethinyl estradiol 0.03 mg and levonorgestrel 0.15 mg) for 90 days. **Figure 1** shows a flow diagram of the study participants.

### Outcome variables, assessment, and follow-up

Before undergoing laparoscopic surgery, the baseline variables of the study participants were recorded, including age, socioeconomic status, occupation, religion, symptoms other than abdominal pain, and VAS scores. The duration of symptoms before enrollment was recorded and should be reported as part of baseline characteristics, as it may influence treatment response. The main outcome was the difference in the degree of endometriosis-related pelvic pain using the visual analog scale (VAS) between baseline and 3 months. This was achieved by taking the recorded symptoms of dyspareunia, dysuria, dyschezia, and pelvic pain among patients in the two groups. Pain caused by endometriosis was measured using the 10-point visual analog scale both before and after 3 months of treatment. To obtain the main outcome, patient assessments were conducted before and 3 months after treatment in both groups.

### Statistical analysis

Data were collected using Microsoft Excel and later moved to OriginPro for statistical analysis, plotting, and imaging. Repeated-measures ANOVA was conducted to assess changes in VAS score over time. Corrections for multiple comparisons were performed using the Bonferroni correction method. Multivariable linear regression analysis was additionally performed to identify predictors of pain reduction. Statistical significance was set at p < 0.05.

## Results

Of the 220 recruited participants, those who met the inclusion criteria were assigned to two study groups: 108 to group I and 112 to group II. Furthermore, almost 80 participants were lost to follow-up or discontinued the intervention in both groups; hence, 70 participants per group were included in the outcome analyses. **Figure 1** shows a flowchart of participants’ admission and adherence to treatment. The median age of the study participants in groups I and II was 33.56 and 33.17 years, respectively. The results showed no significant difference in participants’ age, suggesting a homogeneous distribution across the study groups.

Furthermore, the socio-demographic and clinical characteristics of the study participants showed no significant differences in the endometriosis-associated symptoms (**Table 1**). The present study showed that the largest number of participants were homemakers, accounting for 52.8% in the OCP group and 54.2% in the dienogest group. Workers contributed 28.5% in both groups, followed by students at 18.5% in levonorgestrel-containing COC and 17.2% in dienogest. The data showed that the largest number of participants fell into the lower-income slab, accounting for 52.8% in the OCP group and 54.2% in the dienogest group. The middle-income slab contributed 28.5% in both groups, followed by the upper-income group at 18.5% in levonorgestrel-containing COC and 17.2% in dienogest.

**Table 1:** Sociodemographic and clinical characteristics of study participants.

Variable	OCP group (n=70)	Dienogest group (n=70)	p-value
Age (years)	31.47 ± 8.90	31.08 ± 8.78	0.794
BMI (kg/m²)	23.31 ± 2.10	24.11 ± 3.20	0.082
Age at menarche (years)	12.61 ± 1.24	12.48 ± 1.18	0.541
Menstrual cycle duration (days)	28.94 ± 2.33	29.21 ± 2.18	0.463
Chronic pelvic pain, n (%)	70 (100)	70 (100)	—
Dysmenorrhea, n (%)	47 (67.1)	49 (70.0)	0.711
Dyspareunia, n (%)	31 (44.3)	29 (41.4)	0.728
Infertility, n (%)	19 (27.1)	21 (30.0)	0.702
Menstrual irregularities, n (%)	26 (37.1)	24 (34.3)	0.729

**Figure 2:**
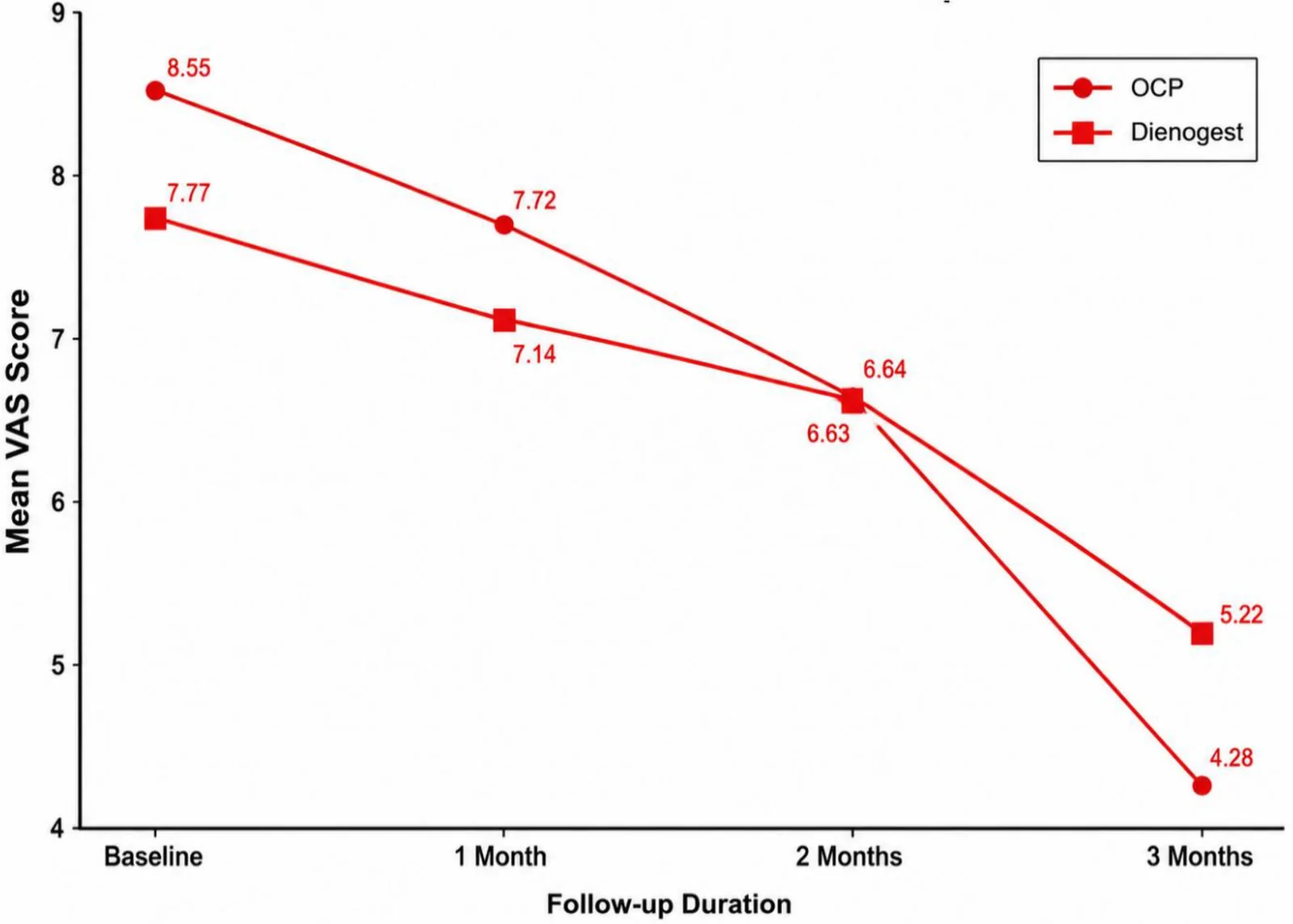
Trends of mean visual analog scale (VAS) score at baseline, 1, 2, and 3 months1-, 2-, and 3-months follow-up in women with endometriosis-associated chronic pelvic pain in Group I and Group II. The mean VAS scores are presented at baseline and after 1, 2, and 3 months of treatment in the dienogest and OCP groups. Lower VAS scores indicate lower pain severity. Differences in pain trajectories between groups over time were evaluated using repeated-measures analysis of variance (ANOVA), followed by Bonferroni-adjusted *post hoc* comparisons, where appropriate.

**Figure 3:**
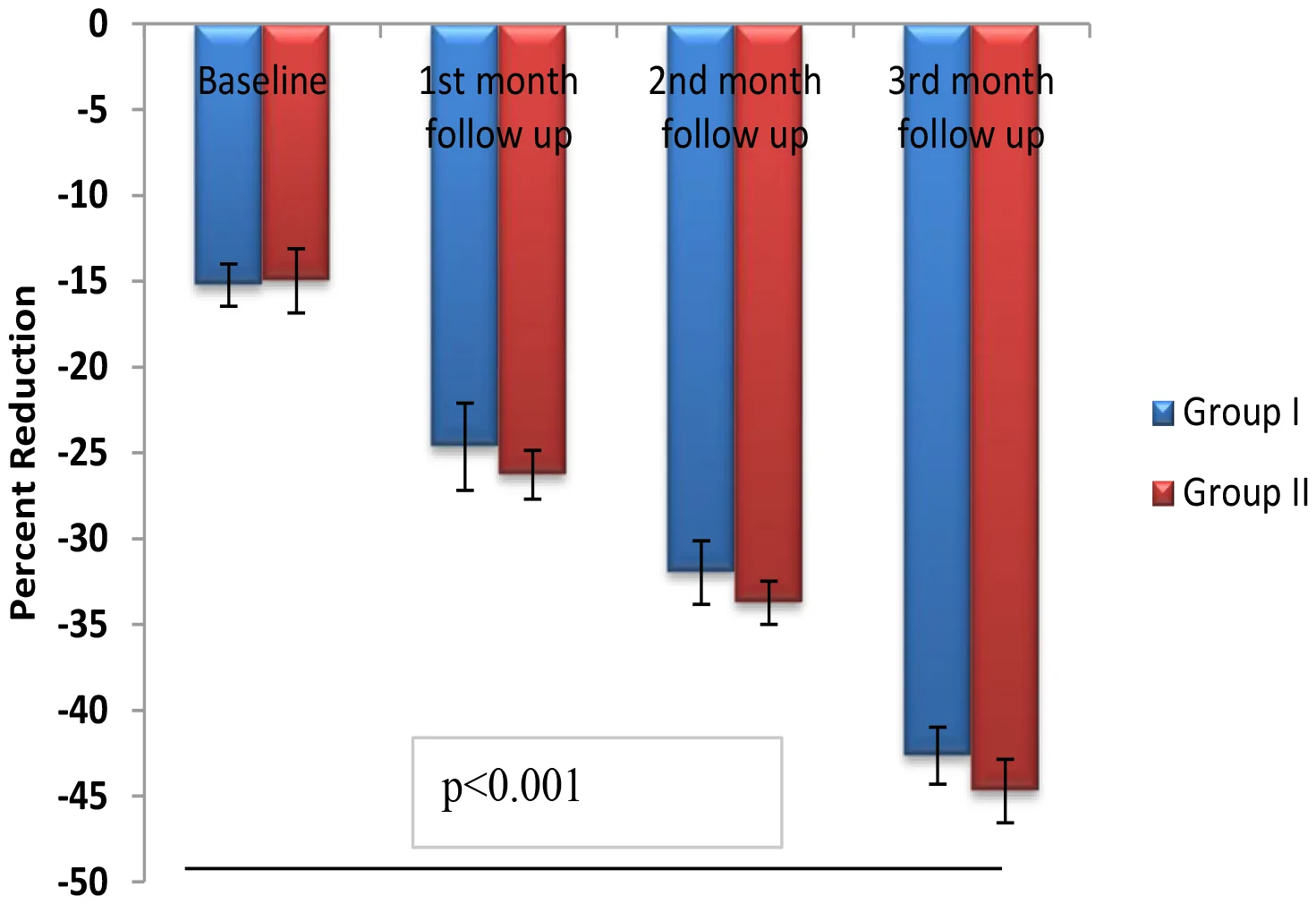
Percentage reduction in endometriosis-associated chronic pelvic pain following treatment with dienogest (2 mg/day) or continuous ethinyl estradiol 0.03 mg/levonorgestrel 0.15 mg combined oral contraceptive (EE/LNG COC). The percentage reduction was calculated as [(follow-up VAS score − baseline VAS score)/baseline VAS score] × 100, with negative values indicating improvement relative to baseline. Bars represent the mean values, and error bars denote the standard error of the mean (SEM).

In this comparative analysis, most participants in group I showed VAS scores in the range of 7–10 before the intervention (92.86%); after the intervention, this shifted to 2–4 in 84.28% of participants, followed by 15.72%. With 4-6 (**Figure 2**). In group II, it was observed that all study participants of group II showed VAS scores in the range of 7–10 before intervention (100%), which after the intervention changed to 2–4 in 58.57% of study participants, followed by 38.57% with 4–6 and 2.86% with 7–10 VAS score (**Figure 3**). In **Figure 3**, the comparative VAS score performance analysis of OCP versus Dienogest showed significant improvement in both groups compared to baseline. Clinically meaningful improvement refers to a ≥30% reduction in VAS score. The proportion of participants achieving a clinically meaningful reduction in pain was compared between groups.

**Figure 4:**
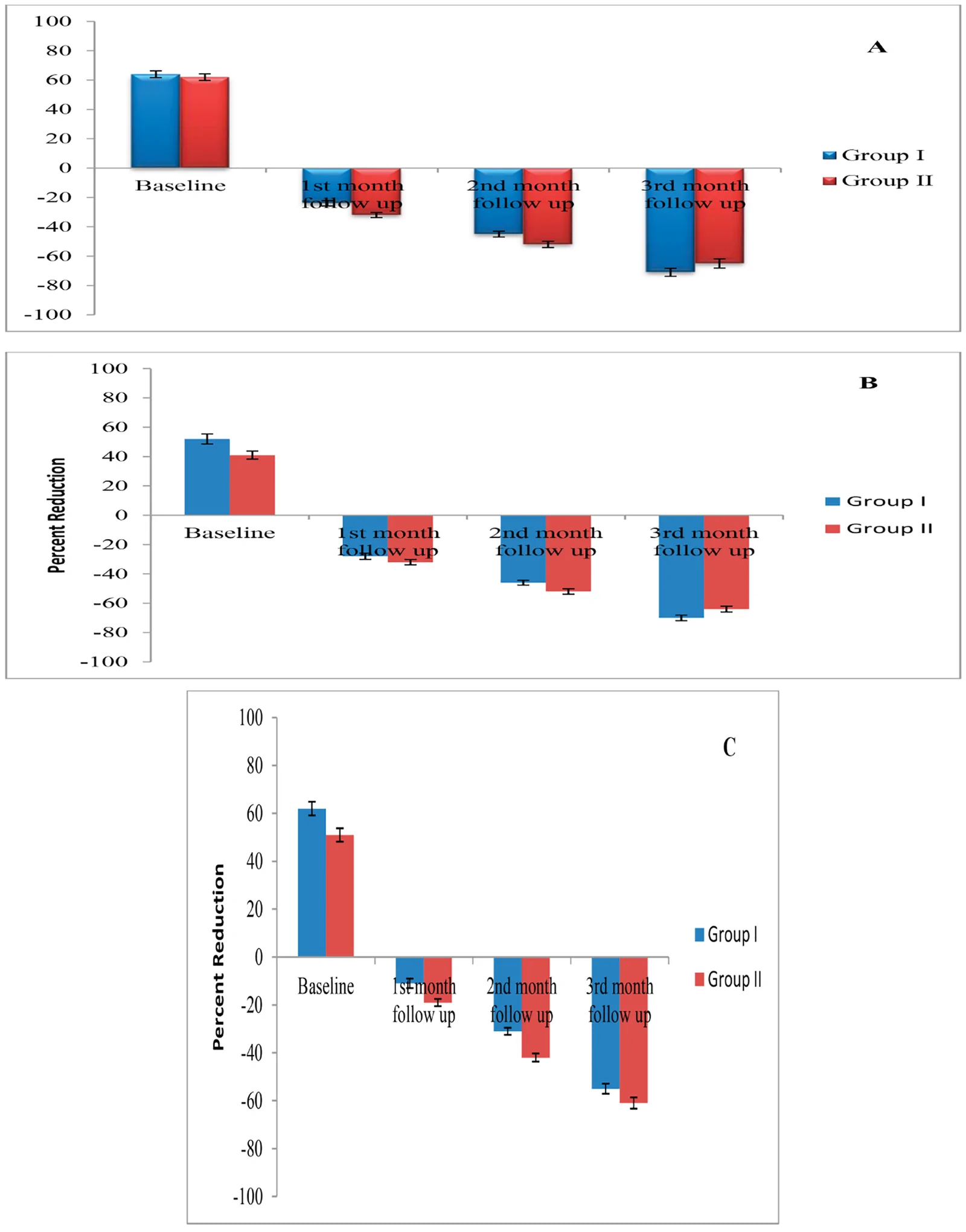
Percentage improvement in **(A)** dysmenorrhea, **(B)** dyspareunia, and **(C)** menstrual irregularities following treatment with dienogest (2 mg/day) or continuous ethinyl estradiol 0.03 mg/levonorgestrel 0.15 mg combined oral contraceptive (EE/LNG COC). The percentage reduction was calculated as [(follow-up score − baseline score)/baseline score] × 100. Bars represent the mean values, and error bars denote the standard error of the mean (SEM).

A significant difference in the percentage of chronic pain associated with endometriosis is shown in **Figure 4** (p<0.001). Follow-up showed a significant reduction in pain intensity compared with baseline; accordingly, levonorgestrel-containing COC and dienogest showed comparable improvement in chronic pain-related complaints (**Figure 4**; **Table 2**). Both therapies demonstrated significant improvement in VAS scores during follow-up compared to baseline. A non-significant reduction in dysmenorrhea, menstrual irregularities, and dyspareunia was observed in patients who received levonorgestrel-containing COC. **Figure 2** shows the intergroup analysis at baseline and at the 1st, 2nd, and 3^rd^ follow-ups, revealing a significant reduction in endometriosis-related symptoms in both groups when follow-ups were compared with baseline recruitment data.

**Table 2:** Longitudinal comparison of visual analog scale (VAS) scores between OCP and dienogest groups during follow-up.

Follow-up duration	OCP group (mean ± SD)	Dienogest group (mean ± SD)	Mean difference	p-value
Baseline	8.55 ± 0.98	7.77 ± 0.90	0.78	0.001
1st Month Follow-up	7.72 ± 0.68	7.14 ± 0.58	0.58	0.003
2nd Month Follow-up	6.64 ± 0.77	6.63 ± 0.87	0.01	0.921
3rd Month Follow-up	4.28 ± 1.15	5.22 ± 1.66	-0.94	<0.001
Mean Reduction in VAS Score	4.27 ± 1.32	2.55 ± 1.41	1.72	<0.001

**Figure 5:**
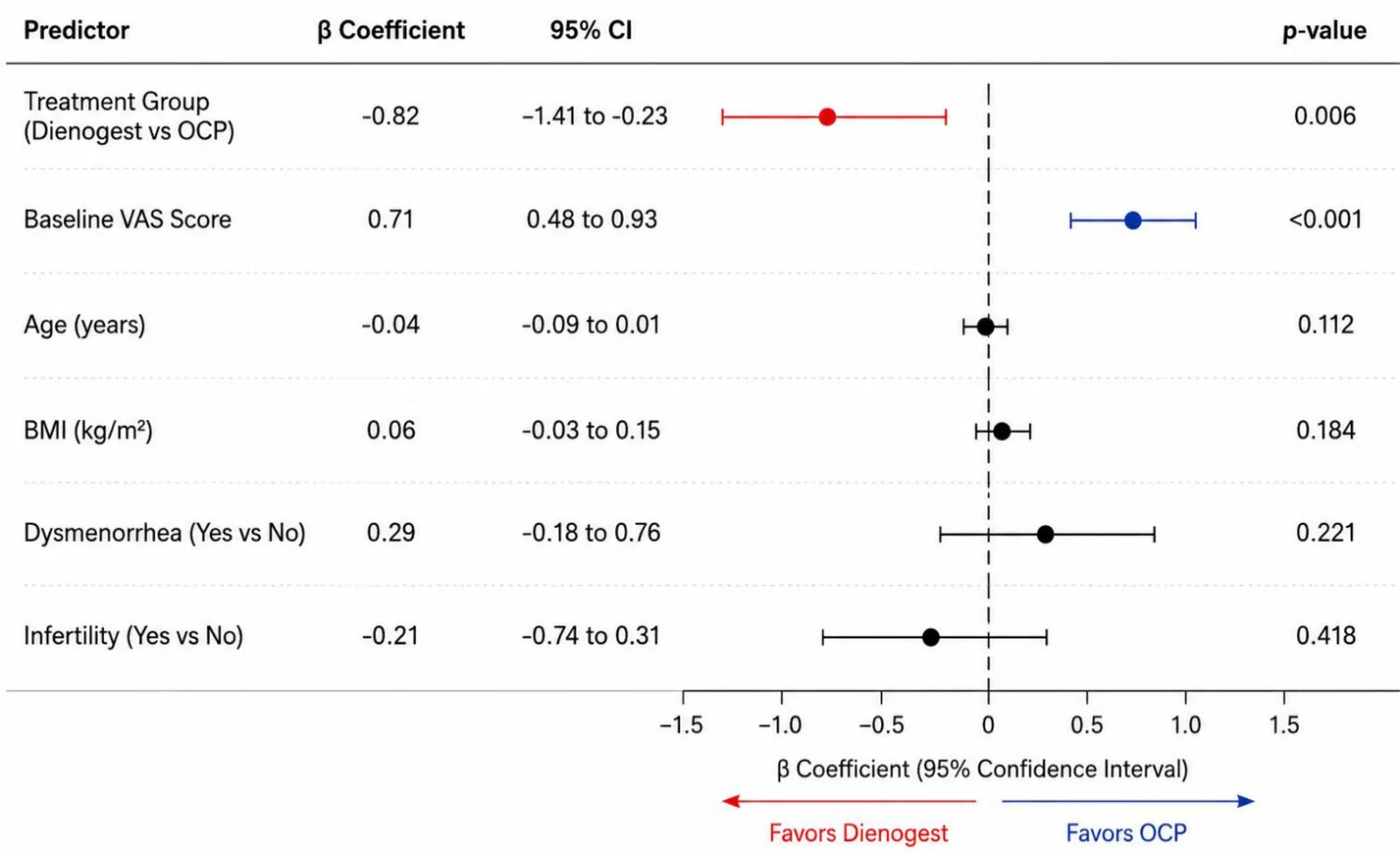
Multivariable linear regression analysis for predictors of reduction in VAS score at 3 months. Forest plot showing β coefficients with corresponding 95% confidence intervals (CIs) for the variables included in the multivariable linear regression model. The vertical dashed line represents a null effect (β = 0). Negative β coefficients indicate greater pain reduction associated with dienogest, whereas positive coefficients favor OCP treatment. Statistical significance was determined using multivariable linear regression.

**Figure 6:**
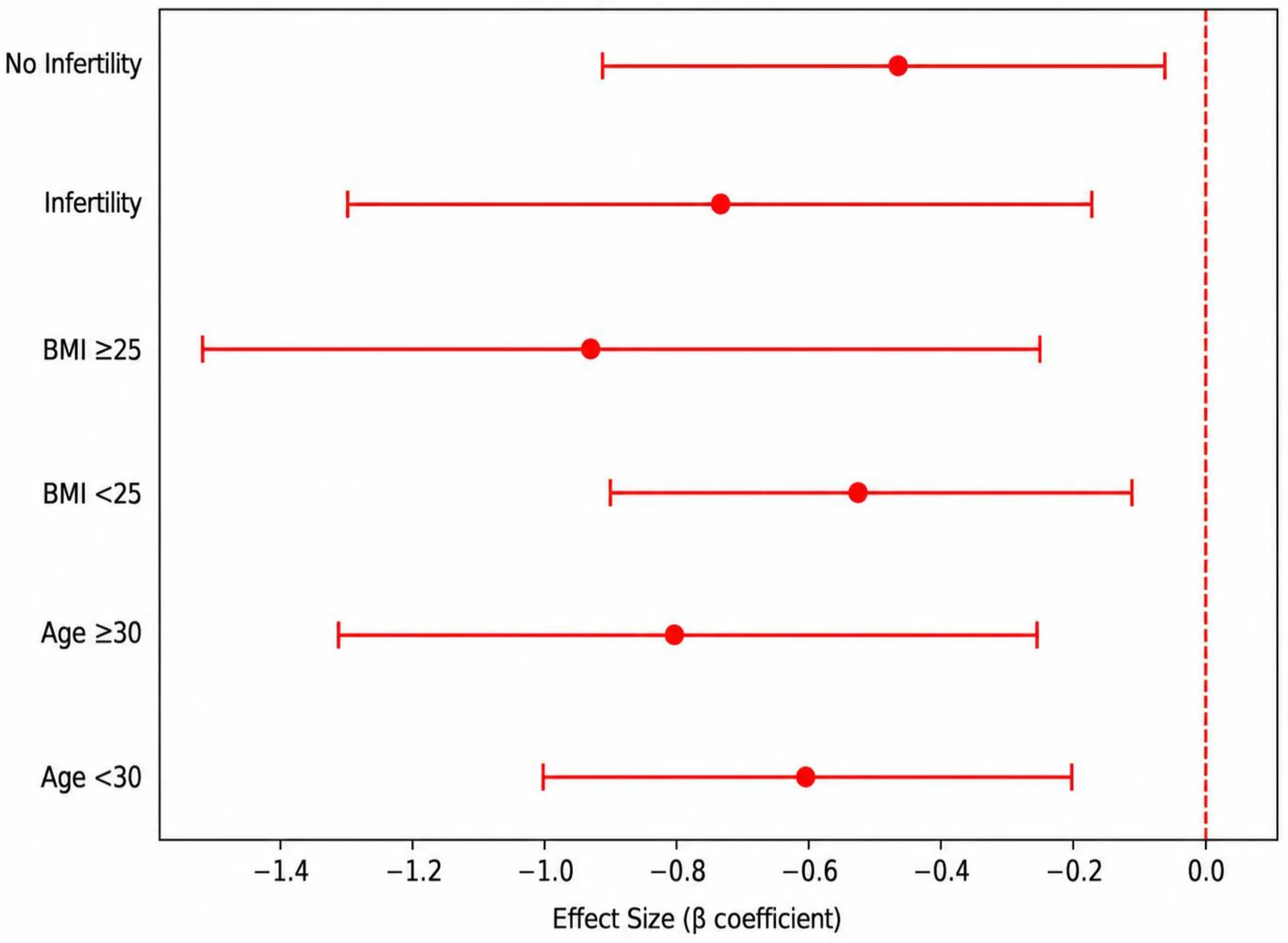
Forest plot subgroup analysis. Effect sizes (β coefficients) with 95% confidence intervals (CIs) are shown for predefined subgroups according to age, body mass index (BMI), and infertility status. The vertical reference line represents no treatment effect (β = 0). Subgroup effects were estimated using interaction terms in the multivariable regression model.

Repeated-measures ANOVA demonstrated a significant reduction in the VAS scores over time in both treatment groups (time effect: p < 0.001). Intergroup comparison demonstrated significantly greater pain reduction in the OCP group at the 3-month follow-up. Multivariate regression analysis demonstrated that the treatment group and baseline VAS score were independent predictors of pain reduction at 3 months (**Table 3**; **Figure 5**). The baseline VAS score was an independent predictor of pain reduction at 3 months, and the treatment group remained associated with the outcome after adjustment for baseline pain severity and other covariates. Participants receiving OCP therapy demonstrated a significantly greater reduction in VAS scores than those in the dienogest group after adjusting for age, BMI, dysmenorrhea, infertility status, and baseline pain severity. Clinically meaningful improvements in chronic pelvic pain, dysmenorrhea, dyspareunia, and menstrual irregularities were observed in both treatment groups. No statistically significant intergroup differences were observed in the improvement of secondary symptoms (**Table 4**). A significantly greater proportion of participants in the OCP group achieved clinically meaningful pain reduction compared to the dienogest group during follow-up (82.9% vs. 65.7%, p=0.019) (**Table 5**). Subgroup analysis demonstrated a consistent reduction in the VAS scores across all evaluated demographic and clinical subgroups. A greater reduction in pain severity was observed among participants aged ≥30 years and those with a BMI ≥25 kg/m² (**Table 6**; **Figure 6**).

**Table 3:** Multivariable linear regression analysis for predictors of reduction in VAS score at 3 months.

Predictor variable	Beta coefficient (β)	95% confidence interval	p-value
Treatment Group (Dienogest vs OCP)	-0.82	-1.41 to -0.23	0.006
Age (years)	-0.04	-0.09 to 0.01	0.112
BMI (kg/m²)	0.06	-0.03 to 0.15	0.184
Baseline VAS Score	0.71	0.48 to 0.93	<0.001
Dysmenorrhea	0.29	-0.18 to 0.76	0.221
Infertility	-0.21	-0.74 to 0.31	0.418

**Table 4:** Symptom improvement following hormonal therapy during follow-up.

Clinical symptom	OCP group improvement (%)	Dienogest group improvement (%)	p-value
Chronic pelvic pain	70.0	64.0	0.284
Dysmenorrhea	42.65	44.71	0.816
Dyspareunia	71.0	65.0	0.432
Menstrual irregularities	55.0	61.0	0.471

**Table 5:** Clinically meaningful response analysis (≥30% reduction in VAS Score).

Response category	OCP group (n=70)	Dienogest group (n=70)	p-value
Achieved ≥30% VAS Reduction	58 (82.9%)	46 (65.7%)	0.019
Did Not Achieve ≥30% Reduction	12 (17.1%)	24 (34.3%)	

**Table 6:** Subgroup analysis of mean reduction in VAS scores.

Subgroup	Mean reduction in VAS score	95% CI	p-value
Age <30 years	0.62	0.21–1.02	0.004
Age ≥30 years	0.81	0.33–1.29	0.001
BMI <25 kg/m²	0.53	0.14–0.91	0.008
BMI ≥25 kg/m²	0.91	0.27–1.54	0.005
Infertility	0.71	0.18–1.24	0.011
No infertility	0.49	0.06–0.92	0.027

**Figure 7:**
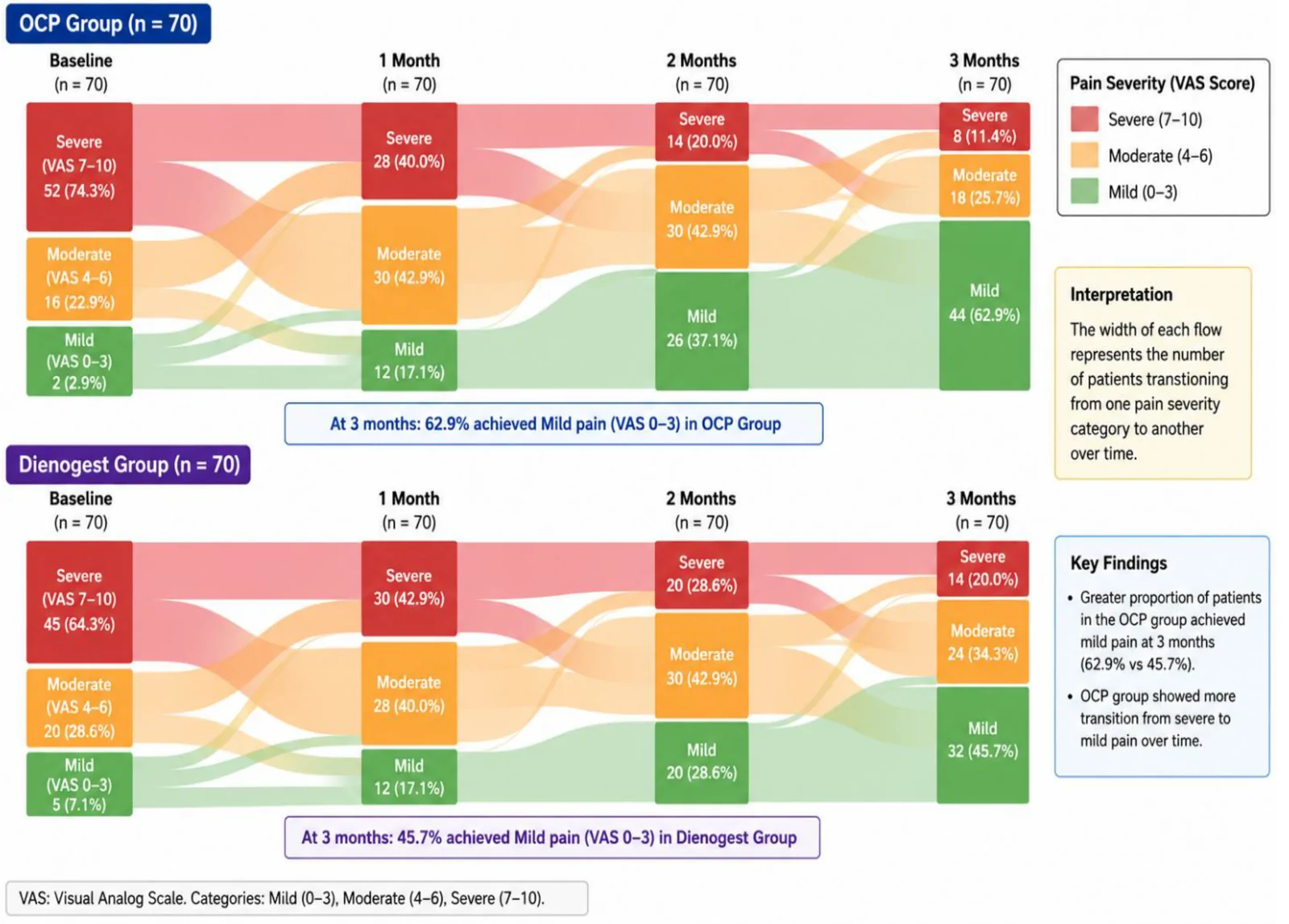
Sankey plot demonstrating longitudinal transition in pain severity during hormonal therapy for endometriosis. The diagram depicts changes in pain severity categories (mild, moderate, and severe) from baseline to 1, 2, and 3-month follow-up in the Group I and Group II. The width of each flow is proportional to the number of participants transitioning between categories over time. Pain severity categories were defined according to VAS scores (mild: 0–3, moderate: 4–6, severe: 7–10).

The Sankey plot demonstrates a progressive reduction in pain severity over time in both treatment groups (**Figure 7**). A greater proportion of participants in the OCP group transitioned from the severe and moderate pain categories to mild pain severity during follow-up than that in the dienogest group. At 3 months, 62.9% of the participants in the OCP group had mild pain compared with 45.7% in the dienogest group. These findings suggest substantial longitudinal improvement in symptom burden following hormonal therapy, with a comparatively greater pain reduction observed in the OCP group (**Figure 7**).

**Figure 8:**
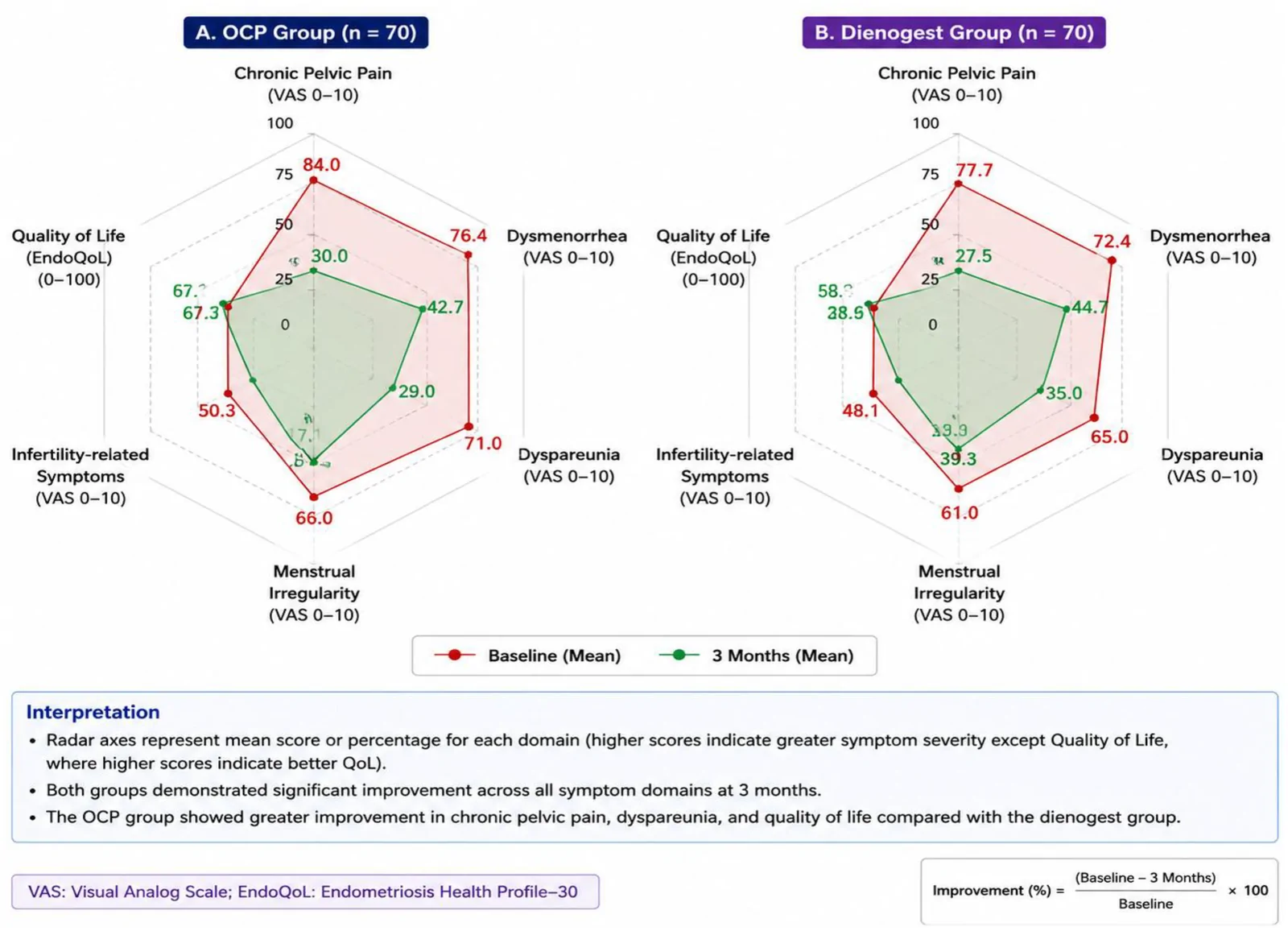
Radar plot demonstrating multidimensional symptom improvement following hormonal therapy in women with endometriosis. Radar plots compare mean baseline and 3-month scores for chronic pelvic pain, dysmenorrhea, dyspareunia, menstrual irregularity, infertility-related symptoms, and quality of life (EndoQoL). Higher scores indicate greater symptom severity, except for quality-of-life scores, where higher values represent better quality of life. The mean values are presented for each domain.

**Figure 9:**
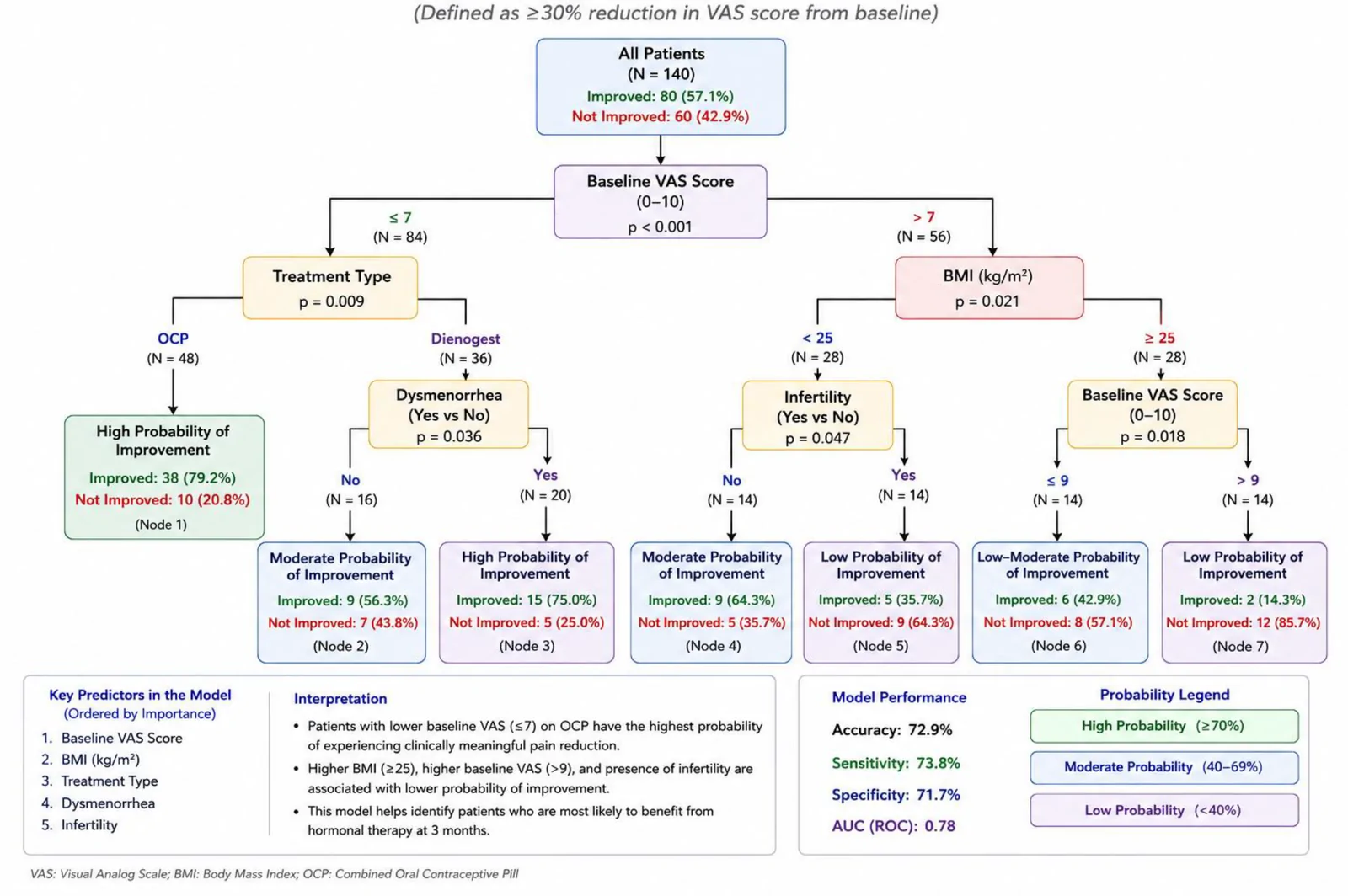
Decision tree model for predicting clinically meaningful pain reduction following Hhormonal therapy in women with endometriosis. Clinically meaningful improvement was defined as a ≥30% reduction in the VAS score from baseline after 3 months of treatment. The classification tree identified the baseline VAS score, treatment type, body mass index, dysmenorrhea, and infertility status as the principal predictors of treatment response. Terminal nodes displayed the probability of clinically meaningful improvement, while the overall model performance was summarized by accuracy, sensitivity, specificity, and area under the receiver operating characteristic curve (AUC).

The radar chart revealed substantial multidimensional improvement in terms of symptoms after hormonal therapy in the two treatment groups (**Figure 8**). There was progressive improvement in chronic pelvic pain, dysmenorrhea, dyspareunia, menstrual irregularities, and infertility symptoms compared to the baseline values. The OCP group had more improvement in terms of chronic pelvic pain and dyspareunia compared to the other group; however, overall, there was a significant reduction in the severity of the symptoms in both groups (**Figure 8**). The exploratory decision tree analysis found that baseline VAS score, treatment regimen, body mass index, dysmenorrhea, and infertility were the variables significantly correlated with achieving a clinically relevant decrease in pain after 3 months of treatment. The model was found to have moderate discriminatory capacity (accuracy 72.9%, sensitivity 73.8%, specificity 71.7%, AUC 0.78). With the small sample size and lack of external validation, the results may be viewed more as hypothesis-generating than definitive. The decision tree analysis showed that baseline VAS score was the best predictor of a clinically significant decrease in pain at the 3-month follow-up (**Figure 9**). The participants with baseline VAS scores less than or equal to 7 who underwent OCP treatment had the greatest chances of improvement, with 79.2% of participants demonstrating clinically significant pain reduction (**Figure 9**).

In contrast, participants with baseline VAS scores >9 and BMI ≥25 kg/m² had the lowest probability of improvement. Dysmenorrhea and infertility status further influenced treatment response trajectories in the intermediate-risk groups. Overall, the predictive model demonstrated good discriminative ability for identifying patients who are most likely to benefit from hormonal therapy.

## Discussion

This prospective observational cohort study compared the clinical effectiveness of dienogest and continuous combined oral contraceptive pills (Levonorgestrel-containing COC) in women with endometriosis-associated chronic pelvic pain. This study compared the outcomes of women who received dienogest or continuous combined oral contraceptive therapy according to routine clinical decision-making and patient preferences, without randomization. Both treatment arms demonstrated significant reductions in pain severity and overall symptom burden at the 3-month follow-up visit. In contrast, participants receiving the evaluated levonorgestrel/ethinyl estradiol combined oral contraceptive regimen demonstrated a greater reduction in VAS scores than those receiving dienogest during the 3-month follow-up period (13). In addition to a significant decrease in chronic pelvic pain, there was marked improvement in dysmenorrhea, dyspareunia, and menstrual abnormalities in both groups of patients (14). It can be concluded that both hormonal therapies were associated with clinically meaningful improvement in endometriosis-associated symptoms. However, in the present observational cohort study, the evaluated levonorgestrel/ethinyl estradiol combined oral contraceptive regimen demonstrated a greater short-term reduction in pain severity than dienogest. Because the treatment allocation was not randomized, these findings should be interpreted as observed associations rather than definitive evidence of superiority. It

The hormonal and anti-inflammatory effects of the medications used may explain these observed therapeutic effects. Endometriosis is characterized by estrogen-dependent growth, progesterone resistance, increased aromatase activity, and chronic inflammation in the ectopic endometrium (15). The mechanism of action of levonorgestrel-containing COC includes suppression of ovulation and decreased menstrual bleeding, resulting in lower estrogen levels and reduced prostaglandin-mediated inflammation (16). Dienogest is a progestogen with anti-inflammatory and antiproliferative properties. It has been shown to suppress local estradiol production, prevent angiogenesis, reduce cytokine expression, and induce decidualization and atrophy of endometriotic implants. All these effects may explain the greater reduction in VAS in patients receiving dienogest (17).

These results are consistent with earlier observations and randomized controlled trials that have shown significant improvements in pelvic pain and dysmenorrhea with dienogest treatment. 19. Del Forno et al. observed that both dienogest and estrogen-based hormone therapies were effective in managing pain associated with endometriosis; however, dienogest had a superior response in certain cases. Similarly, Chen et al. found positive clinical responses in a real-world cohort who received dienogest treatment (18). According to a systematic review (Brown et al.), the use of ethinyl estradiol/levonorgestrel COC continues to be relevant as a first-line treatment for dysmenorrhea and pelvic pain caused by endometriosis (5). The present study adds to the existing body of literature by including comparative prospective data from Indian women.

There are various clinical implications of these findings, as endometriosis is a chronic, recurrent disease that requires long-term symptom management. Both continuous levonorgestrel-containing COC and dienogest were found to be effective in managing endometriosis symptoms (19). Thus, they may be considered potential conservative therapies for patients who do not require surgical management. Previous hormonal therapy may influence symptom severity and treatment responsiveness in women with endometriosis. Because detailed information regarding prior medical treatment was not collected systematically, residual confounding related to previous therapeutic exposure cannot be excluded. Future prospective studies should record previous hormonal therapies and account for them in baseline adjustment or propensity-score matching. The levonorgestrel-containing combined oral contraceptive evaluated in this study was associated with substantial short-term pain reduction and represents a relatively inexpensive conservative treatment option Levonorgestrel-containing COC (20). While the exploratory decision tree analysis revealed several possible predictors of effectiveness, including initial pain intensity, treatment regimen, body mass index, dysmenorrhea, and infertility, the results must be considered carefully. The comparatively small sample size and the absence of internal or external validation contribute to the possibility of overfitting and reduce the generalizability. Thus, the model can be used only for generating hypotheses but not for making a clinically applicable prediction model. Further multicenter research with more participants and external validation is needed before implementing it in the clinical setting. In the present prospective observational cohort, the OCP group experienced a higher reduction of pain; however, since the treatment was not randomized, it means that this finding can be regarded as an association but not as a superiority. Both therapies led to a significant relief from pain due to endometriosis, and a higher short-term reduction was found in the OCP group in this cohort. Since only one combined oral contraceptive formulation (ethinyl estradiol 0.03 mg/levonorgestrel 0.15 mg) was investigated, the present findings should not be generalized to other ethinyl estradiol/levonorgestrel COC containing different progestins, such as drospirenone or cyproterone acetate, which may differ in pharmacological profile and clinical efficacy (21, 22). Comparative studies evaluating multiple COC formulations are warranted. Because endometriosis is a chronic and recurrent disease, assessment of treatment effectiveness requires long-term follow-up. The present study evaluated outcomes over a 3-month period and therefore reflects only short-term clinical effectiveness. Whether the observed differences in pain reduction between the evaluated treatment regimens are maintained over longer follow-up periods remains uncertain. Future prospective studies with follow-up extending beyond 6–12 months are needed to evaluate sustained pain control, recurrence, treatment adherence, and long-term safety.

## Limitations

Nevertheless, this study had several limitations that need to be considered when evaluating the results. First, it is necessary to note that this study was conducted using a single-center, observational cohort design, which may raise concerns about external validity and residual confounding. Second, the lack of histopathological confirmation of endometriosis, as this study relied on clinical and ultrasonographic methods, which may have led to misclassification. Another potential limitation of this study is the significant number of participants who were lost to follow-up, which may introduce attrition bias. Moreover, the limited follow-up time precluded evaluation of recurrence and long-term safety outcomes. Because ovarian reserve markers were not assessed, the study could not account for possible baseline differences in ovarian reserve among younger participants with endometriosis. Because endometriosis was not confirmed histopathologically in all cases, and other causes of chronic pelvic pain, such as fibroids, adhesions, or pelvic infection, may not have been completely excluded, residual misclassification bias cannot be ruled out. We acknowledge that the high attrition rate may have introduced attrition bias and may limit the generalizability of the findings. ASRM staging was not available for all participants because routine laparoscopic staging was not performed; therefore, differences in disease severity between groups cannot be fully excluded. Differences in symptom duration, age at onset, and unmeasured disease severity may have influenced treatment response, and these factors were not fully controlled for, which may reduce internal validity. Because standardized disease severity classification was not available for all participants, residual confounding by disease extent cannot be excluded. Baseline imbalance in pain severity between groups may have influenced the magnitude of improvement, despite adjustment in multivariable analysis. Repeated-measures ANOVA was applied to longitudinal VAS data; however, formal testing of all model assumptions was not performed. This is now acknowledged as a methodological limitation. Adverse events, menstrual bleeding patterns, and treatment discontinuation due to side effects were not systematically recorded; therefore, safety outcomes could not be evaluated comprehensively. The decision tree model was developed using a relatively small sample without internal cross-validation or external validation. Therefore, the predictive performance may be optimistic, and the model should be considered exploratory. Validation in larger, independent cohorts is necessary before clinical implementation. Another limitation is that previous hormonal treatment history, including prior exposure to GnRH agonists, progestogens, and combined oral contraceptives, was not systematically recorded. Consequently, baseline comparability with respect to previous medical therapy could not be evaluated, and residual confounding related to prior treatment exposure cannot be excluded. Quality of life, fertility outcomes, lesion staging, and long-term adverse effects were not assessed. Despite these limitations, the study offers important insights related to hormonal therapy for endometriosis-related pain. Further multicenter, longitudinal studies with large sample sizes and longer follow-up periods, standardized imaging or histopathological confirmation, and quality-of-life and reproductive outcome measures are needed. Another limitation is that only a single combined oral contraceptive formulation (ethinyl estradiol 0.03 mg/levonorgestrel 0.15 mg) was evaluated. Therefore, the findings cannot be generalized to all combined oral contraceptive formulations because different progestins may have different therapeutic effects on endometriosis-associated pain. Moreover, the 3-month follow-up period was relatively short for a chronic and recurrent disease such as endometriosis. Consequently, the study could not evaluate the durability of pain relief, symptom recurrence, long-term analgesic efficacy, treatment adherence, or long-term safety. Therefore, the findings should be interpreted as representing short-term clinical outcomes rather than long-term comparative effectiveness.

## Strengths of the study

The current study has some significant advantages. First, the prospective observational study design enabled longitudinal assessment of symptom course and treatment response across multiple follow-up visits. Second, pain severity was assessed at multiple time points using the visual analog scale (VAS), a sensitive tool for measuring changes in endometriosis-associated pain intensity. Third, comparing two popular hormone regimens increases the external validity in a real-world setting. Evaluation of important secondary endpoints, such as dysmenorrhea, dyspareunia, and menstrual irregularities, provided a more holistic assessment of the treatment effect than pain severity alone. The study also provides valuable comparisons for the Indian population, where prospective real-world comparisons of the efficacy of dienogest versus COC regimens are limited. Lastly, although an observational design was chosen, baseline characteristics of participants were matched across groups, thereby increasing the internal validity of outcome comparisons.

## Conclusion

In addition, the intervention using levonorgestrel/ethinyl estradiol combined oral contraceptive Levonorgestrel-containing COC in women experiencing endometriosis-related pain led to improvement in both pain and unpleasant symptoms. There was a considerable improvement in unpleasant symptoms in the intragroup comparison at follow-up compared with baseline, with reductions in inflammation and ovulation induced by ER receptor inhibition in endometriotic tissues. Clinically significant improvement was noted in pelvic pain associated with endometriosis in both the dienogest and combination oral contraceptive pill groups. Both treatment regimens were associated with significant improvements in endometriosis-associated pain. In this prospective observational cohort study, the evaluated levonorgestrel/ethinyl estradiol combined oral contraceptive regimen was associated with a greater short-term reduction in VAS scores than dienogest. These findings reflect short-term clinical outcomes, and longer-term prospective studies are required to determine whether these treatment effects are maintained over time.

## Data Availability

The original contributions presented in the study are included in the article/supplementary material. Further inquiries can be directed to the corresponding author.
